# Editorial: Circadian rhythms and cancer hallmarks: toward advances in immune-based therapeutics, and outcomes

**DOI:** 10.3389/fonc.2025.1571086

**Published:** 2025-02-28

**Authors:** Elizabeth Cash, Pasquale F. Innominato, Sandra E. Sephton

**Affiliations:** ^1^ Department of Otolaryngology-Head and Neck Surgery and Communicative Disorders, University of Louisville School of Medicine, Louisville, KY, United States; ^2^ UofL Health-Brown Cancer Center, Louisville, KY, United States; ^3^ Oncology Department, Ysbyty Gwynedd, Betsi Cadwaladr University Health Board, Bangor, United Kingdom; ^4^ Cancer Chronotherapy Team, Warwick Medical School, University of Warwick, Coventry, United Kingdom; ^5^ Research Unit, ‘Chronotherapy, Cancers and Transplantation’, Faculty of Medicine, Paris-Saclay University, Villejuif, France; ^6^ Department of Psychology, Brigham Young University, Provo, UT, United States

**Keywords:** circadian, cancer, immuno-oncology, clock gene, cancer hallmarks, chronotherapy, cancer therapeutics

## Introduction

Circadian rhythms are the daily fluctuations in physiological processes that govern cell cycles and the timing of behaviors (1). Cancer hallmarks are the cellular properties that drive the perennial growth, survival and spread of cancerous cells (2). The disruption of circadian rhythms can contribute to the development of some cancers by affecting the expression of cancer hallmarks (3). Circadian rhythms also temporally regulate cellular immunity, which has important implications for the burgeoning field of immune-based therapeutics (4). Cancer patients exhibiting disrupted circadian rhythms tend to suffer from accelerated tumor growth and metastasis, worse anti-cancer treatment outcomes, and poorer health-related quality of life and overall survival (5). This Research Topic aims to help elucidate the impact of circadian rhythms on processes associated with cancer and its treatments, particularly immunotherapy. We were pleased to accept 11 original manuscripts for this Research Topic.

## Molecular processes

Using a variety of various approaches, several groups have explored the molecular underpinnings of the connection between the circadian clock and cancer. Pan et al. extensively reviewed the role of E-box-binding transcription factors in cell physiology and cancer biology, along with their potential as novel therapeutic targets. The broad group of E-box-binding transcription factors includes two core clock genes, BMAL-1 and CLOCK. This work advances our knowledge of potential therapeutic targets in cancer treatment. Zheng et al. focused on the latest evidence implicating circadian rhythm disruption as a causal factor in endometrial cancer and further explored the potential for mediation of these effects by non-coding RNAs. New research implicating irregular expression of circadian-linked ncRNAs in endometrial cancer cells is described, which may have implications for future targeted therapeutic strategies. Meng et al. deployed a Mendelian randomization analytical approach to large international databases to explore the impact of five genetically independent circadian features on colorectal cancer risk. Strikingly, the authors observed that an individual’s chronotype can significantly contribute to the lifetime risk of developing colorectal cancer. Peng et al. offered a multi-gene prognostic model developed using circadian genes that demonstrated predictive performance for gynecologic cancer prognosis. This bioinformatics approach – validated with human data – also provides insights into potential immunotherapy targets by elucidating immune signaling pathways associated with high-risk circadian gene profiles.

## Clinical relationships

Several groups have critiqued or provided data suggesting the importance of sleep and circadian influences on cancer risk and outcomes. Gouldthorpe et al., reviewing studies that incorporate objective circadian rhythm measurement, provided a useful compendium of the various indices used to summarize circadian endocrine data, actigraphy data, and sleep-wake cycles, with an urgent call for standardization of measurement. The need for standardization was also highlighted by Jagielo et al., who found that both cortisol dysregulation and abnormal rest-activity rhythms are clearly linked with psychological comorbidities in advanced cancer patients, such as pain, fatigue, nausea, vomiting, and cachexia. Promising data on chronomodulated chemotherapy and circadian-targeting behavioral interventions are discussed. Nettnin et al. highlighted a new and promising approach to treatment that regulates circadian function in gliomas, pointing to the importance of targeting circadian regulation in the tumor microenvironment and the distinct need for research in pediatric cancers. Burch et al. explored the association between formally diagnosed sleep disorders and cancer occurrence in a robust sample of Veterans. They identified an optimal sleep duration for protection against oncogenesis: elevated lifetime cancer risk was noted in those who slept on average less than 6, or more than 8, hours per night. Interestingly, both greater severity and longer duration of sleep disorders showed an impact on cancer incidence. Finally, Cash et al. reported on pilot data that open avenues for further exploration of the links between diurnal cortisol expression, head and neck cancer progression, and the potential role of inflammation. They emphasize the importance of multi-day cortisol sampling. Taken together, these data suggest that by recognizing the timing of treatment in relation to cortisol levels, clinicians could optimize treatment schedules to align with patients’ circadian rhythms, potentially enhancing therapeutic outcomes.

## Immunotherapy

Through their unique mechanism of action, immune checkpoint inhibitors are influenced by host physiology, including circadian rhythms. Two mini reviews summarized the growing evidence of interactions between host physiology and checkpoint inhibitors. In the first, Hughes et al. critically revised how light exposure, physical exercise and diet, and notably their respective timing over 24 hours, can impact immunotherapy efficacy in patients with cancer. Balachandran et al. reviewed important emerging data suggesting that sleep disturbance is inversely correlated with tumor response to immunotherapy. Nascent links to the microbiome as a mechanism for these effects are considered along with remaining unanswered questions, such as whether these interconnections can be exploited to improve patient response to immunotherapy. These reviews lay the foundations for novel therapeutic avenues with potential circadian-based lifestyle modifications that could be implemented to manipulate cancer immune responsiveness and maximize the benefit of immunotherapy (6).

## Conclusion

This Research Topic highlights circadian effects on tumor outcome from two perspectives: that of the host (cancer patient) and that of the tumor and its associated microenvironment. These two perspectives offer unique research questions. To improve immunotherapy outcomes, we must establish the mechanisms of circadian effects on tumor growth and acquisition of cancer hallmarks. This line of inquiry can lead to behavioral and pharmacotherapeutic interventions to improve immunotherapy efficacy.

Research must elucidate the molecular clock function in tumors, and how tumors disrupt circadian rhythmicity within their microenvironment. Host and tumor circadian disruptions are rarely studied within the same organism. Such data will inform on bidirectional effects in host–tumor circadian relationships: e.g., do patients with disrupted rhythms have tumors that suppress or promote their own circadian genes? Research imperatives include establishing the temporal precedence of circadian disruption in the development of cancer, whether bidirectional pathways of circadian regulation exist between host and tumor, and a focus on circadian rhythm disorders in malignant and nonmalignant clinical populations. Standardization of protocols for the assessment of popular measures (e.g., cortisol, melatonin) is needed in addition to the pursuit of lesser-studied measures (e.g., core body temperature, pupillometry, blood pressure dipping). One notable area ripe for inquiry regards the effects of sleep and circadian disruption in cancer treatment efficacy (e.g., immunotherapy). We should leverage biorepositories and epidemiological-level circadian data. Despite the need for more research, clear clinical implications include the need for healthcare providers to assess and treat sleep and circadian disruption in cancer patients. While new technologies for measuring, for example, clock gene expression, are promising, many protocols remain potentially burdensome for patients. There remains an urgent need for translation of circadian measurement into clinical cancer settings to inform individualized clinical interventions.
